# Reliability and Validation Study of Advance Care Planning Practice Scale among Health and Social Care Professionals

**DOI:** 10.3390/healthcare12020200

**Published:** 2024-01-15

**Authors:** Sok-Leng Che, Sok-Man Leong, Wing-Sze Lau, Kuai-In Tam

**Affiliations:** 1 Nursing and Health Education Research Centre, Kiang Wu Nursing College of Macau, Macao SAR, China; shirley@kwnc.edu.mo; 2 Education Department, Kiang Wu Nursing College of Macau, Macao SAR, China; lsm@kwnc.edu.mo; 3 Lar de Cuidados de Ká Hó da Federação das Associações dos Operários de Macau, Estr. de Nossa Sra. de Ka Ho, Macao SAR, China

**Keywords:** advance care planning, ACP Practice Scale, ACP practice, health and social care professionals

## Abstract

Advance care planning (ACP) is a crucial process in clinical practice, enabling individuals to articulate their care preferences and goals, with significant implications for future healthcare. However, ACP practice of health and social care professionals (HSCPs) concerning patients, or their family members are rarely explored. The objective of the study was to adapt and validate a four-item scale assessing ACP practices of HSCPs toward patients or their family members. The ACP Practice Scale was evaluated through a cross-sectional online survey administered to HSCPs in Macao, assessing its factor structure, validity, and reliability. Based on a sample of 186 valid responses, the ACP Practice Scale demonstrated satisfactory levels of validity and reliability among HSPCs in Macao. The four-item scale explained 65.87% of the variance in ACP practice and exhibited strong internal consistency, with Cronbach’s alpha and McDonald’s omega coefficients of 0.82. Furthermore, item factor loadings ranged from 0.71 to 0.90. The ACP Practice Scale provides reliable and valid measurements of ACP practice among HSCPs. This instrument can help to enhance our understanding of ACP practices in clinical settings and support the advancement of advance care planning.

## 1. Introduction

Advance care planning (ACP) is a critical process allowing individuals to specify health and medical preferences in the event that they lose their decision-making capacity, which takes into consideration their personal values, goals, and priorities [1,2]. This process involves patients, family members, and health and social care professionals (HSCPs) [3]. Research has demonstrated that the implementation of ACP is associated with the enhancement of patient autonomy, quality of life, quality of care, psychological well-being, and the alignment between patients’ care preferences and the care they receive [4,5,6,7,8]. Furthermore, ACP is found to be positively correlated with reduced utilization of aggressive treatments and hospitalizations [4], allowing individuals’ health and medical preferences to be exercised even when they lose their decision-making capacity.

A study in Hong Kong found that professional advice was a crucial determinant in predicting the preference for Advance Directives (ADs), with family members relying on expert advice regarding healthcare decisions [9]. More than 80 percent of people in Macao believe that it is necessary to discuss end-of-life care with healthcare professionals. This is a reflection of the public’s desire to communicate with healthcare professionals about their healthcare decisions [10]. Contrarily, HSCPs exhibited limited participation in the implementation of ACP. The diminished involvement could be attributed to the lack of confidence to initiate ACP conversations and a deficit in knowledge concerning ACP [11,12]. 

Despite HSCPs being responsible for initiating ACP and facilitating discussions with patients and their designated family members [13,14], as mentioned, the implementation of ACP in daily practice is not as prevalent [15]. In Asian countries, HSCPs have been found to have limited involvement and delayed initiation of ACP [16]. In mainland China, for example, research has shown that fewer than 14% of nursing staff have discussed ACP with patients or family members, but 80% of those nursing staff are willing to take part in discussions of ACP for patients in the future [17].

Given that end-of-life care emphasizes maintaining patients’ quality of life according to their specified preferences, ACP plays a pivotal role in ensuring the provision of high-quality end-of-life care. Several countries and regions, such as the United States, Taiwan, Hong Kong, etc., have enacted different types of laws, and legal documents such as ADs, in an effort to protect patients’ interests. However, in areas without ADs legislation, ACP plays a particularly important role, as it allows family members and healthcare providers to understand patients’ wishes through the communication process of ACP, ensuring patients receive care that meets their needs. While previous studies have examined ACP behaviors, the majority of ACP scales primarily assess the behaviors of patients [18,19,20]. HSCPs’ ACP behavior toward patients or members of their families is rarely examined. In Smith et al. [21]’s study, 16 items were used to evaluate physicians’ practice of ACP in the last month. Another study by Gaspar et al. [22] examined the frequency of end-of-life communication by asking ten questions. Nevertheless, neither of these instruments have been validated, and they contain questions unrelated to ACP.

Despite efforts to target healthcare professionals in some studies, a disparity exists in the focus of measures, with some directed at patients with specific diseases [23] or addressing ADs [12,24,25]. The same situation applies to the scales related to the Chinese community. Although the scale was designed for nursing staff, the subjects in the items were not focused. The contents of the scale in Chen’s study [17] include communication between nursing staff and patients or family members, as well as whether nursing staff themselves make their own advanced care plans. Also, the measurement tools lack validation. There are a variety of intervention programs designed to enhance the ACP initiation of HSCPs [19], with some utilizing the completion of ADs as a measurement of successful ACP intervention [6,26]. Nevertheless, this approach may not be viable in jurisdictions where ADs lack legal recognition, such as Macao and Mainland China.

The iterative and interactive nature of ACP discussions requires HSCPs to take a proactive role [27]. While these discussions may not always lead to the completion of ADs or the designation of a surrogate decision maker, they remain crucial for effective communication among patients, their families, and healthcare providers [28]. Therefore, the aim of this study was to adapt and validate a scale that assesses the ACP practice exhibited by HSCPs toward patients or their family members. The validation of this scale serves as a foundation for further exploration into the level of engagement of HSCPs in the ACP process.

## 2. Materials and Methods

### 2.1. Study Population and Design

The target population of this cross-sectional online survey was registered medical professionals and social workers working in hospitals, long-term care facilities, and day care centers in Macao during the data collection period.

### 2.2. Scale Adaptation

The items in the ACP Practice Scale were adapted from questionnaires developed by Chen et al. [17] and Hsieh et al. [29]. The scale consists of four items which aim to gauge respondents’ ACP practices over the past 6 months. These items included the following: (1) I have discussed ACP with terminally ill patients or their relatives; (2) I have followed up ACP with terminally ill patients; (3) I have discussed palliative or hospice care with terminally ill patients or their relatives; and (4) I have discussed with terminally ill patients on appointing one surrogate decision maker. Participants were presented with three options for each item: “Yes” (2 points), “No, but I want to do it now or in the future” (1 point), “No, I do not want to do it now nor in the future” (0 point). Approvals have been gained from the authors, for the use in this study.

Following item adaptation, an expert panel was convened to review the items. Three experts from the field of palliative and end-of-life care were invited to the panel, with one from Beijing and two from Macao. In this study, the content validity index (CVI) was used to determine whether items required revision or elimination. Using the scale content validity index (S-CVI) and the item content validity index (I-CVI), items with an I-CVI less than 0.8 were suggested for revision or elimination [30,31]. The experts rated items on a scale from 1 to 4 (completely irrelevant to completely relevant). According to the review of the expert panel, the S-CVI and I-CVI of ACP practices both yielded a score of 1.0, indicating high validity. A recommendation was made to separate the questions for patients and relatives in item 1; however, considering the scale’s intent to measure the practice of ACP rather than the target recipient, and the presence of “or” in the item, the research team decided to leave the content of question 1 unchanged.

To examine the feasibility of the scale and to identify other potential alternative expressions in the target population, a pilot study was conducted in October 2022 prior to the main survey. A sample size of 22 from the population of interest is sufficient [32]. Through the acquaintances of the research team, participants were recruited through a purposeful recruitment process. Twenty-seven participants aged 23 to 58 years (32.85 ± 7.64) were recruited in the pilot test. The Cronbach’s alpha of the scale was 0.64. The final version of the scale remained the same as the original version.

### 2.3. Instruments and Participant Recruitment

Data was collected between November 2022 and May 2023. According to Pett et al. [33]’s recommendation, the sample size should be ten respondents per item, resulting in a minimal recruitment target of at least 40 participants for this study. Convenience and snowball sampling methods were used for sampling through online advertisements and social media platforms. In addition to poster distribution, a short description of the study was provided along with a link to the questionnaire, containing the ACP Practice Scale and demographic characteristics. The information was distributed through various medical and social care professionals’ associations, and social media platforms such as Facebook, WhatsApp, and WeChat. Interested participants could access the questionnaire through a provided link, where they could finish providing informed consent before proceeding with the questionnaire.

### 2.4. Statistical Analysis and Scale Evaluation

The data were summarized using descriptive statistics such as mean, standard deviation, frequency, and percentage. Item analysis was conducted to examine the quality of the items. For dimensionality and internal consistency analysis, an initial exploratory factor analysis was conducted using random selection of approximately 50% of the samples (*n* = 83) from the full dataset, followed by confirmatory factor analysis to verify that the factor structure matched the EFA results using the remaining samples (*n* = 103).

The dimensionality of EFA was determined by extracting factors with eigenvalues greater than 1 and employing principal component analysis (PCA) without rotation. A factor loading exceeding 0.4 was deemed acceptable [34]. The suitability of the data for factor analysis (PCA) was assessed by ensuring a Kaiser–Meyer–Olkin (KMO) measurement above 0.70 and a significant result (*p* < 0.05) in Bartlett’s test of sphericity. Additionally, parallel analysis (PA) was conducted to compare the eigenvalues [33]. The study assessed the internal consistency through the utilization of Cronbach’s alpha, McDonald’s omega, and composite reliability (CR), while also examining the average variance extracted (AVE) to analyze convergent validity. Models with CR values greater than 0.7 and AVE values greater than 0.5 were deemed satisfactory [35]. 

Confirmatory factor analysis (CFA) was conducted using the maximum likelihood method. The adequacy and acceptability of the model were determined based on meeting the following criteria: comparative fit index (CFI) > 0.9, non-norm-fitting index (Tucker–Lewis Index, TLI) > 0.9, root mean square error of approximation (RMSEA < 0.08), and standardized root mean square residual (SRMR) < 0.09 [36]. All data were coded utilizing Microsoft Office Excel 2013 and analyzed employing IBM Statistical Package for the Social Sciences for Windows Version 22 (SPSS, version 22). Additionally, Amos (version 22.0) was utilized to conduct CFA.

### 2.5. Ethical Approval

The study received ethical approval from the Research Management and Development Department of Kiang Wu Nursing College of Macau (reference: REC-2022.502). All participants were informed about the objectives of the study and their right to withdraw from the study at any time. Informed consent was secured from all participants who agreed to participate in this study.

## 3. Results

### 3.1. Participants’ Characteristics

**Figure 1:**
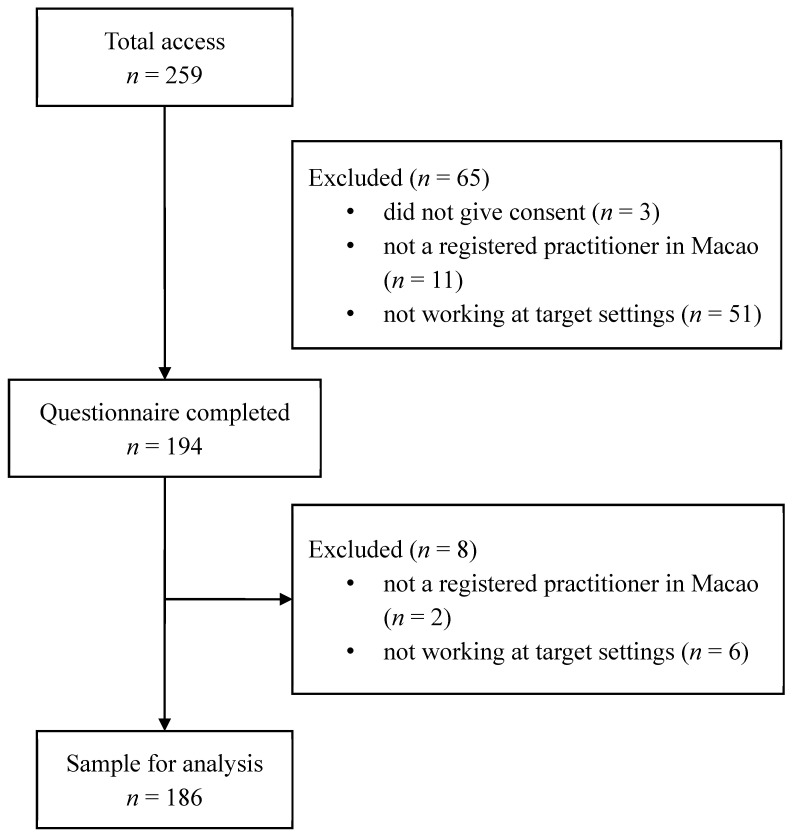
Flowchart of the sample size for analysis.

The questionnaire was completed by 194 of the 259 people who accessed the questionnaire page, with 186 providing valid responses. The reasons for exclusion included the following: (1) did not give consent to participate (*n* = 3); (2) not a registered medical professional nor social worker in Macao (*n* = 13); and (3) not working in a hospital, long-term care facility, or day care center (*n* = 57), with some of them working in clinics or schools (Figure 1).

The study included a predominantly female sample (83.9%). Most of the participants were aged between 31 and 45 years (50.0%). The age range of the participants was 23 to 61 years, with a mean age of 34.5 ± 8.2. Two-thirds of the participants held a bachelor’s degree (66.7%). Half of them were married (50.5%) and did not have children (54.3%). The majority of the participants reported having no religious beliefs (81.2%). In terms of occupation, the majority of the participants were nurses (69.9%) with 6 to 15 years of professional experience (48.9%) (ranged 0 to 37 years; 10.8 ± 7.9). The participants primarily worked in hospitals (57.0%).

Most of the participants worked in organizations that had not implemented ACP (78.0%), and a significant proportion of the participants (79.0%) reported no previous experience with ACP training (79.0%). The average case load in the past month was 36.1 (*SD* = 48.6) (Table 1).

### 3.2. Item and Factor Analysis

The item discrimination test showed positive discrimination for all items (Table 2). The KMO was 0.749, and Bartlett’s test of sphericity was statistically significant ($x_{6}^{2}\;$= 132.266; *p* < 0.001), indicating that the matrix was suitable for factor extraction. PA also supported a single factor model. The items accounted for 65.87% of the total variance. Furthermore, the Cronbach’s alpha coefficient for the scale was 0.82, and the Omega coefficients yielded similar results (*ω* = 0.82), indicating a high level of internal consistency.

**Figure 2:**
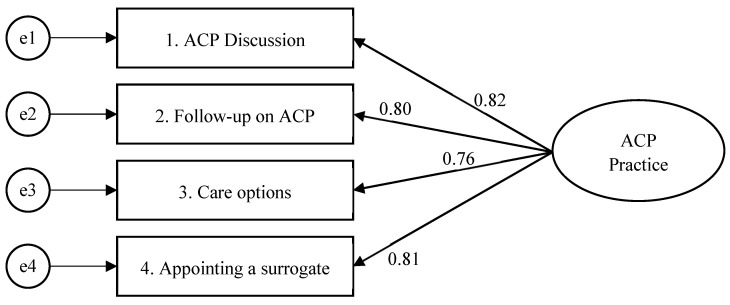
Structural equation model for the fitting model of the ACP Practice Scale (*n* = 103).

The results of the CFA Indicate that the factor structure of the ACP Practice Scale was well-fitted by the model. The model fit indices for the factor structure of the scale were as follows: $x_{2}^{2}$= 10.291 (*p* = 0.006), CFI = 0.959, TLI = 0.878, RMSEA = 0.202 (90% C.I. = 0.092–0.331), and SRMR = 0.034. Additionally, the scale demonstrated sufficient convergent validity, as evidenced by a CR of 0.88 and AVE of 0.66 (Table 3). The factor loading of the items ranged from 0.71 to 0.90. The path diagram illustrating the CFA model is shown in Figure 2.

## 4. Discussion

The ACP Practice Scale demonstrated a satisfactory result of reliability and validity among HSCPs in Macao, except for a low RMSEA. Although a sample size of 10 samples per 1 item represents sufficient analyses [33], the number is insufficient to ensure robustness of the results due to the small number of items [37]. There are many cutoff values for RMSEA to suggest a good or bad fitting model [36,37]. However, most of the simulation studies use relatively large degrees of freedom (*df*) and large sample sizes [38,39]. The literature suggests that there is a tendency for RMSEA to indicate a poor fitting model when the *df* and sample size are small due to greater sampling error [40,41]. For example, in MacCallum et al. [37]’s simulation, the minimum sample size is 3488 when *df* = 2 in order to fit the RMSEA = 0.05. Considering the ACP Practice Scale is a single-factor model with four indicators, we therefore inferred that the small *df* and small sample size likely contribute to the low RMSEA. It is recommended not to calculate RMSEA for low *df* models but to identify the specification error instead [40].

Regarding the items, the ACP Practice Scale emphasizes the process of communication. In a city without legalized ADs, such as Macao, HSCPs lack clear guidelines to adhere to. The specific role and responsibilities of HSCPs in ACP communication remain ambiguous. Additionally, there is a lack of statistical data pertaining to the completion rates of end-of-life care plans, both within hospital settings and long-term care facilities. Given the nascent state of ACP practice, the items included in the ACP Practice Scale serve to underscore the importance of comprehending patients’ preferences.

The items of the scale involve both patients and their family members, as in many cases, patients are unconscious or unable to make medical decisions, such as people with severe dementia, requiring family members to act as surrogate decision makers [42,43]. Furthermore, healthcare providers and family members often overlook the ability of patients, particularly in Chinese communities [44]. Therefore, appointing a surrogate decision maker is important not only because it reduces family conflicts, but also because it ensures that the patient’s wishes are followed when care decisions are made. Surrogate decision makers need guidance and experience through multiple ACP discussions to alleviate anxiety and obtain reassurance when they are making decisions that align with the patient’s expectations [45]. HSCPs have the potential to offer such support to family members throughout patient care [46].

ACP behavior can reflect the communication process of HSCPs, rather than the results of AD completion only. Previous studies indicated that patients and their family members hold the expectation that HSCPs will take the initiative to engage in ACP discussions and acknowledge their preferences [42,47]. In order to provide high-quality end-of-life care, healthcare organizations must acknowledge the importance of ACP and ensure their staff possess the necessary skills to facilitate personalized ACP conversations [48]. Nevertheless, the items of the scale address only the actual practice of ACP, without taking into consideration the given opportunity to the participants to be involved in ACP practice, nor examining their competency or the quality of their practice. Since these factors may affect ACP practice, it is recommended that consideration be given to associated factors when using the scale. For example, HSCPs’ knowledge of ACP, skills to implement ACP, organizational desire to implement ACP, follow-up appointments, documentation of ACP discussions, and patient/family members satisfaction with the end-of-life care received, etc.

The ACP Practice Scale underwent a revision within the Chinese cultural framework, with its content focusing specifically on the ACP practice of professionals toward patients and their families. The scale offers organizations the opportunity to assess the performance of their employees in the field of palliative care. In addition, the results of the scale can serve as evidence and as a basis for establishing training arrangements, as well as facilitate the improvement of palliative care services based on the findings. Moreover, this scale can be used as an effective measurement tool in related research, addressing the issue of combining healthcare professionals’ ACP behaviors with those of patients and their family members, which is prevalent in the existing scales. 

There are some limitations in this study. Firstly, since the number of items in the scale is small, it is recommended that future research adopts a larger sample size for more accurate psychometric analysis to increase the credibility of the results. Moreover, other psychometric analyses such as discriminant validity and test–retest reliability were not implemented in this study. Further analysis should be conducted in future studies. Most of the study participants were nurses, whereas other healthcare professionals made up a smaller percentage. Furthermore, the frequency of participants caring for patients with life-limiting illnesses was not addressed in the study. Given that certain specialties encounter a greater number of patients with life-limiting illnesses, this could be a contributing factor to the practice of ACP. Future studies could consider including such variables for a more in-depth analysis. Therefore, it is imperative to exercise caution when generalizing the findings. Lastly, the potential for reporting bias may arise due to the self-reporting nature of the scale. To enhance interpretation in future studies, it is recommended to incorporate both objective and subjective measures.

## 5. Conclusions

The process of communication is the core of ACP. The measurement of communication behaviors could provide insight into HSCPs’ engagement with ACP. The ACP Practice Scale is a tool that focuses on communication behaviors between health and social care professionals and patients and their family members. It addresses the issue that there is a lack of clarity in terms of the targets and content in existing tools. The initial validation of the ACP Practice Scale showed that it is a tool with good reliability and validity. The measurement tool is particularly suitable for measuring professionals’ ACP practice toward patients or families in cities or countries that have not yet legalized ADs.

## Figures and Tables

**Table 1:** Participants’ characteristics (*n* = 186).

Variables	*n*	%
Gender		
Male	30	16.1
Female	156	83.9
Age (year)		
≤30	71	38.2
31–45	93	50.0
≥46	22	11.8
Education level		
Bachelor’s degree	124	66.7
Postgraduate diploma	21	11.3
Master’s degree or above	41	22.0
Marital status		
Not married	79	42.5
Married/cohabited	94	50.5
Separated/divorced	12	6.5
Widowed	1	0.5
Children		
No	101	54.3
Yes	85	45.7
Religious belief		
No	151	81.2
Yes	35	18.8
Profession		
Nurse	130	69.9
Doctor	10	5.4
Therapist	12	6.5
Social worker	34	18.3
Professional experience (year)		
≤5	55	29.6
6–15	91	48.9
16–25	27	14.5
≥26	13	7.0
Organization		
Hospital	106	57.0
Community/day care center	41	22.0
Long-term care facility	39	21.0
ACP practice in the current organization		
No	145	78.0
Yes	41	22.0
ACP Training		
No	147	79.0
Yes	39	21.0

**Table 2:** Item and distribution analysis of the ACP Practice Scale (*n* = 83).

Item	Mean	*SD*	Skewness	Item Discrimination	Cronbach’s α if Item Deleted	Corrected Item-Total Correlation
1. I have discussed ACP with terminally ill patients or their relatives	1.10	0.48	0.26	−5.50 ***	0.74	0.71
2. I have followed up ACP with terminally ill patients	1.12	0.50	0.23	−6.06 ***	0.71	0.77
3. I have discussed palliative or hospice care with terminally ill patients or their relatives	1.35	0.57	−0.18	−15.12 ***	0.83	0.53
4. I have discussed with terminally ill patients on appointing one surrogate decision maker	1.08	0.47	0.27	−4.43 ***	0.80	0.59
Total	4.65	1.64			0.82	

*** *p* < 0.001. Items were scored 0 to 3.

**Table 3:** Exploratory factor analysis and convergent validity of the ACP Practice Scale (*n* = 83).

	Factor Loading	Communalities
1. I have discussed ACP with terminally ill patients or their relatives	0.86	0.74
2. I have followed up ACP with terminally ill patients	0.90	0.81
3. I have discussed palliative or hospice care with terminally ill patients or their relatives	0.71	0.50
4. I have discussed with terminally ill patients on appointing one surrogate decision maker	0.77	0.59
Eigenvalues	2.64	
% of Variance	65.87	
% of Cumulative Variance	65.87	
Cronbach’s alpha (*α*)/McDonald’s omega (*ω*)	0.82/0.82	
Composite Reliability (CR)	0.88	
Average of variance extracted (AVE)	0.66	

## Data Availability

The data presented in this study are available on request from the corresponding author.

## References

[1] Hospital Authority (2019). HA Guidelines on Advance Care Planning. https://www.ha.org.hk/visitor/ha_visitor_index.asp?Content_ID=252688&Lang=ENG&Dimension=100&Parent_ID=200776&Ver=HTML.

[2] Rietjens J.A.C., Sudore R.L., Connolly M., van Delden J.J., Drickamer M.A., Droger M., van der Heide A., Heyland D.K., Houttekier D., Janssen D.J.A. (2017). Definition and recommendations for advance care planning: An international consensus supported by the European Association for Palliative Care. Lancet Oncol..

[3] Vanderhaeghen B., Bossuyt I., Opdebeeck S., Menten J., Rober P. (2017). Toward Hospital Implementation of Advance Care Planning: Should Hospital Professionals Be Involved?. Qual. Health Res..

[4] Brinkman-Stoppelenburg A., Rietjens J.A., van der Heide A. (2014). The effects of advance care planning on end-of-life care: A systematic review. Palliat. Med..

[5] Detering K.M., Hancock A.D., Reade M.C., Silvester W. (2010). The impact of advance care planning on end of life care in elderly patients: Randomised controlled trial. BMJ.

[6] Houben C.H.M., Spruit M.A., Groenen M.T.J., Wouters E.F.M., Janssen D.J.A. (2014). Efficacy of Advance Care Planning: A Systematic Review and Meta-Analysis. J. Am. Med. Dir. Assoc..

[7] McMahan R.D., Tellez I., Sudore R.L. (2020). Deconstructing the Complexities of Advance Care Planning Outcomes: What Do We Know and Where Do We Go? A Scoping Review. J. Am. Geriatr. Soc..

[8] Schichtel M., Wee B., Perera R., Onakpoya I. (2020). The Effect of Advance Care Planning on Heart Failure: A Systematic Review and Meta-analysis. J. Gen. Intern. Med..

[9] Chu L.-W., Luk J.K., Hui E., Chiu P.K., Chan C.S., Kwan F., Kwok T., Lee D., Woo J. (2011). Advance directive and end-of-life care preferences among Chinese nursing home residents in Hong Kong. J. Am. Med. Dir. Assoc..

[10] Leong S.M., Tam K.I., Che S.L., Zhu M.X. (2021). Prevalence and Predictors of Willingness to Make Advance Directives among Macao Chinese. Int. J. Environ. Res. Public Health.

[11] Gilissen J., Pivodic L., Smets T., Gastmans C., Stichele R.V., Deliens L., Block L.V.D. (2017). Preconditions for successful advance care planning in nursing homes: A systematic review. Int. J. Nurs. Stud..

[12] Gilissen J., Pivodic L., Dael A.W.-V., Cools W., Stichele R.V., Block L.V.D., Deliens L., Gastmans C. (2020). Nurses’ self-efficacy, rather than their knowledge, is associated with their engagement in advance care planning in nursing homes: A survey study. Palliat. Med..

[13] Piers R., Albers G., Gilissen J., De Lepeleire J., Steyaert J., Van Mechelen W., Steeman E., Dillen L., Berghe P.V., Block L.V.D. (2018). Advance care planning in dementia: Recommendations for healthcare professionals. BMC Palliat. Care.

[14] Wichmann A.B., van Dam H., Thoonsen B., Boer T.A., Engels Y., Groenewoud A.S. (2018). Advance care planning conversations with palliative patients: Looking through the GP’s eyes. BMC Fam. Pract..

[15] Knight T., Malyon A., Fritz Z., Subbe C., Cooksley T., Holland M., Lasserson D. (2020). Advance care planning in patients referred to hospital for acute medical care: Results of a national day of care survey. eClinicalMedicine.

[16] Martina D., Lin C.P., Kristanti M.S., Bramer W.M., Mori M., Korfage I.J., van der Heide A., van der Rijt C.C., Rietjens J.A. (2021). Advance Care Planning in Asia: A Systematic Narrative Review of Healthcare Professionals’ Knowledge, Attitude, and Experience. J. Am. Med. Dir. Assoc..

[17] Chen Y., Cheng Q., Wang Y., Liu X., Li X., Mao T., Peng J. (2019). Knowledge-attitude-practice and counter-measures of Advance Care Planning among nurses. Chin. Nurs. Manag..

[18] Houben C.H.M., Spruit M.A., Luyten H., Pennings H.-J., Boogaart V.E.M.v.D., Creemers J.P.H.M., Wesseling G., Wouters E.F.M., Janssen D.J.A. (2019). Cluster-randomised trial of a nurse-led advance care planning session in patients with COPD and their loved ones. Thorax.

[19] Lum H.D., Barnes D.E., Katen M.T., Shi Y., Boscardin J., Sudore R.L. (2018). Improving a Full Range of Advance Care Planning Behavior Change and Action Domains: The PREPARE Randomized Trial. J. Pain Symptom Manag..

[20] Sævareid T.J.L., Thoresen L., Gjerberg E., Lillemoen L., Pedersen R. (2019). Improved patient participation through advance care planning in nursing homes—A cluster randomized clinical trial. Patient Educ. Couns..

[21] Smith T.A., Kim M., Piza M., Davidson P.M., Clayton J.M., Jenkins C.R., Ingham J.M. (2014). Specialist respiratory physicians’ attitudes to and practice of advance care planning in COPD. A pilot study. Respir. Med..

[22] Gaspar C., Alfarroba S., Telo L., Gomes C., Bárbara C. (2014). End-of-life care in COPD: A survey carried out with Portuguese Pulmonologists. Rev. Port. Pneumol..

[23] Zhou G., Stoltzfus J.C., Houldin A.D., Parks S.M., Swan B.A. (2010). Knowledge, Attitudes, and Practice Behaviors of Oncology Advanced Practice Nurses Regarding Advanced Care Planning for Patients With Cancer. Oncol. Nurs. Forum.

[24] Gilissen J., Dael A.W.-V., Gastmans C., Stichele R.V., Deliens L., Detering K., Block L.V.D., Pivodic L. (2021). Differences in advance care planning among nursing home care staff. Nurs. Ethics.

[25] Shepherd J., Waller A., Sanson-Fisher R., Clark K., Ball J. (2018). Knowledge of, and participation in, advance care planning: A cross-sectional study of acute and critical care nurses’ perceptions. Int. J. Nurs. Stud..

[26] Liu L., Zhao Y.-Y., Yang C., Chan H.Y.-L. (2021). Gamification for promoting advance care planning: A mixed-method systematic review and meta-analysis. Palliat. Med..

[27] Jimenez G., Tan W.S., Virk A.K., Low C.K., Car J., Ho A.H.Y. (2018). Overview of Systematic Reviews of Advance Care Planning: Summary of Evidence and Global Lessons. J. Pain Symptom Manag..

[28] Aslakson R.A., Isenberg S.R., Crossnohere N.L., Conca-Cheng A.M., Moore M., Bhamidipati A., Mora S., Miller J., Singh S., Swoboda S.M. (2019). Integrating advance care planning videos into surgical oncologic care: A randomized clinical trial. J. Palliat. Med..

[29] Hsieh C.-C., Huang H.-P., Tung T.-H., Chen I.C., Beaton R.D., Jane S.-W. (2019). The exploration of the knowledge, attitudes and practice behaviors of advanced care planning and its related predictors among Taiwanese nurses. BMC Palliat. Care.

[30] Polit D.F., Beck C.T. (2006). The content validity index: Are you sure you know what’s being reported? critique and recommendations. Res. Nurs. Health.

[31] Zamanzadeh V., Ghahramanian A., Rassouli M., Abbaszadeh A., Alavi-Majd H., Nikanfar A.R. (2015). Design and Implementation Content Validity Study: Development of an instrument for measuring Patient-Centered Communication. J. Caring Sci..

[32] Perneger T.V., Courvoisier D.S., Hudelson P.M., Gayet-Ageron A. (2015). Sample size for pre-tests of questionnaires. Qual. Life Res..

[33] Pett M.A., Lackey N.R., Sullivan J.J. (2003). Making Sense of Factor Analysis: The Use of Factor Analysis for Instrument Development in Health Care Research.

[34] Howard M.C. (2015). A Review of Exploratory Factor Analysis Decisions and Overview of Current Practices: What We Are Doing and How Can We Improve?. Int. J. Hum.–Comput. Interact..

[35] Fornell C., Larcker D.F. (1981). Structural Equation Models with Unobservable Variables and Measurement Error: Algebra and Statistics. J. Mark. Res..

[36] Hu L.T., Bentler P.M. (1999). Cutoff criteria for fit indexes in covariance structure analysis: Conventional criteria versus new alternatives. Struct. Equ. Model. A Multidiscip. J..

[37] MacCallum R.C., Browne M.W., Sugawara H.M. (1996). Power analysis and determination of sample size for covariance structure modeling. Psychol. Methods.

[38] Breivik E., Olsson U.H., Cudeck R., Du Toit S., Sorbom D. (2001). Adding variables to improve fit: The effect of model size on fit assessment in LISREL. Structural Equation Modeling: Present and Future.

[39] Kenny D.A., McCoach D.B. (2003). Effect of the Number of Variables on Measures of Fit in Structural Equation Modeling. Struct. Equ. Model. Multidiscip. J..

[40] Kenny D.A., Kaniskan B., McCoach D.B. (2015). The Performance of RMSEA in Models With Small Degrees of Freedom. Sociol. Methods Res..

[41] Lai K., Green S.B. (2016). The Problem with Having Two Watches: Assessment of Fit When RMSEA and CFI Disagree. Multivar. Behav. Res..

[42] Fosse A., Schaufel M.A., Ruths S., Malterud K. (2014). End-of-life expectations and experiences among nursing home patients and their relatives—A synthesis of qualitative studies. Patient Educ. Couns..

[43] Harvey S.V., Adenwala A.Y., Lane-Fall M.B. (2021). Defining Familial Interactions and Networks: An Exploratory Qualitative Study on Family Networks and Surrogate Decision-Making. Crit Care Explor..

[44] Dutta O., Lall P., Patinadan P.V., Car J., Low C.K., Tan W.S., Ho A.H.Y. (2019). Patient autonomy and participation in end-of-life decision-making: An interpretive-systemic focus group study on perspectives of Asian healthcare professionals. Palliat. Support. Care.

[45] Su Y., Yuki M., Hirayama K. (2020). The experiences and perspectives of family surrogate decision-makers: A systematic review of qualitative studies. Patient Educ. Couns..

[46] Becqué Y.N., Rietjens J.A.C., van der Heide A., Witkamp E. (2021). How nurses support family caregivers in the complex context of end-of-life home care: A qualitative study. BMC Palliat. Care.

[47] Hall A., Rowland C., Grande G. (2019). How Should End-of-Life Advance Care Planning Discussions Be Implemented According to Patients and Informal Carers? A Qualitative Review of Reviews. J. Pain Symptom Manag..

[48] Andreassen P., Neergaard M.A., Brogaard T., Skorstengaard M.H., Jensen A.B. (2015). The diverse impact of advance care planning: A long-term follow-up study on patients’ and relatives’ experiences. BMJ Support. Palliat. Care.

